# Strong Negative Interference by Calcium Dobesilate in Sarcosine Oxidase Assays for Serum Creatinine Involving the Trinder Reaction

**DOI:** 10.1097/MD.0000000000000905

**Published:** 2015-06-12

**Authors:** Xiuzhi Guo, Li’an Hou, Xinqi Cheng, Tianjiao Zhang, Songlin Yu, Huiling Fang, Liangyu Xia, Zhihong Qi, Xuzhen Qin, Lin Zhang, Qian Liu, Li Liu, Shuling Chi, Yingying Hao, Ling Qiu

**Affiliations:** From the Department of Laboratory Medicine, Peking Union Medical College Hospital, Chinese Academic Medical Science and Peking Union Medical College (XG, LH, XC, SY, HF, LX, ZQ, XQ, LZ, QL, LL, SC, YH, LQ); and Beijing Hospital, National Center for Clinical Laboratories, Ministry of Health, Beijing, PR China (TZ).

## Abstract

The vasoprotective drug calcium dobesilate is known to interfere with creatinine (Cr) quantifications in sarcosine oxidase enzymatic (SOE) assays. The aim of this study was to investigate this interference in 8 different commercially available assays and to determine its clinical significance.

In *in vitro* experiments, interference was evaluated at 3 Cr levels. For this, Cr was quantified by SOE assays in pooled serum supplemented with calcium dobesilate at final concentrations of 0, 2, 4, 8, 16, 32, and 64 μg/mL. Percent bias was calculated relative to the drug-free specimen. For *in vivo* analyses, changes in serum concentrations of Cr, cystatin C (CysC; a renal function marker), and calcium dobesilate were monitored in healthy participants of group I before and after oral calcium dobesilate administration. In addition, variations in interference were also examined among different SOE assays using serum obtained from healthy participants of group II. Lastly, Cr levels from the 10 patients treated with calcium dobesilate were measured using 4 SOE assays and liquid chromatography-isotope dilution tandem mass spectrometry (LC-IDMS/MS) for comparison.

Our in vitro analyses indicated that the presence of 8 μg/mL calcium dobesilate resulted in a −4.4% to −36.3% reduction in Cr serum concentration compared to drug-free serum for 8 SOE assays examined. In vivo, Cr values decreased relative to the baseline level with increasing drug concentration, with the lowest Cr levels obtained at 2 or 3 hours after drug administration in participants of group I. The observed Cr concentrations for participants in group II were reduced by −28.5% to −3.1% and −60.5% to −11.6% at 0 and 2 hours after administration related to baseline levels. The Cr values of 10 patients measured by Roche, Beckman, Maker, and Merit Choice SOE assays showed an average deviation of −20.0%, −22.4%, −14.2%, and −29.6%, respectively, compared to values obtained by LC-IDMS/MS.

These results revealed a clinically significant negative interference with calcium dobesilate in all sarcosine oxidase-based Cr assays, but the degree of interference varied greatly among the assays examined. Thus, extra care should be taken in evaluating Cr quantification obtained by SOE assays in patients undergoing calcium dobesilate therapy.

## INTRODUCTION

Creatinine (Cr) is crucial for renal function and is the most commonly used index of renal function in patients for both treatment monitoring and prognosis. The traditional alkaline picrate method used to determine Cr concentrations in plasma or serum is known to be subject to interference from numerous endogenous and exogenous substances.^1^ In 2006, the US National Kidney Disease Education Program Laboratory Working Group published its “recommendations” to improve serum Cr analysis.^2^ These guidelines promoted standardization for Cr testing protocols, including the use of enzymatic assays for Cr quantification. Currently, the sarcosine oxidase enzymatic assay (herein after referred to as the “SOE assay”) is the primary method used in medical laboratories due to its sensitivity, specificity, and ability for automated analysis.^3^ According to data from the Chinese National Center for Clinical Laboratories, more than 67% of the 1899 clinical laboratories in China used SOE assays to quantify Cr in 2014, compared with 54% in 2009. However, several substances are reported to interfere with Cr quantification by SOE assay.^4–8^ We previously found significant discrepancies in Cr values in individual samples analyzed by SOE and alkaline picrate, or by different SOE assays, with some values quantified by SOE assay 50% less than the “true” Cr concentration determined by liquid chromatography-isotope dilution tandem mass spectrometry (LC-IDMS/MS). This finding greatly affected opinions regarding the state of illness and resulting clinical treatment. Upon further investigation, we found that the majority of samples with divergent Cr values were obtained from patients undergoing calcium dobesilate therapy, and thus theorized that the false decrease in Cr might be caused by interference from calcium dobesilate.

Calcium dobesilate (calcium 2,5-dihydroxybenzenesulfonate) is a vasoprotectant that can remarkably reduce capillary permeability, blood viscosity, and high platelet activity, as well as improve abnormal hemorheology and microcirculation,^9^ and is widely used to treat diabetic retinopathy,^10^ chronic venous insufficiency,^11^ and various microangiopathies.^12^ Moreover, recent studies have shown its protective effect on diabetic nephropathy^13^ and gentamicin-induced acute kidney injury.^14^

Calcium dobesilate interference in enzymatic Cr methods was first reported by Guder and Hoffmann in 1986;^3^ however, the clinical impact has not yet been reported in detail. Our survey of more than 10 SOE assays commercially available in China yielded only 2 that were unaffected by calcium dobesilate according to their claims. With the great increase in calcium dobesilate administration for kidney-related diseases and the continuous development of the diagnostic industry over the past 20 years, the following questions should be addressed: Does calcium dobesilate universally interfere with current Cr SOE assays? How substantial is the degree of this interference in patients with clinical conditions? What is the clinical significance of this interference with regards to treatment decision-making? In this study, the effect of calcium dobesilate on Cr quantification was analyzed with in vitro exogenous addition and in vivo postadministration experiments to determine its clinical significance in 8 commercially available SOE assays.

## METHODS

### Reagents and Equipment

Eight commercially available Cr SOE assays were evaluated using the following reagent/analyzer combinations routinely used in hospitals: Roche Creatinine Plus reagent/Roche Cobas c702 biochemical analyzer (F. Hoffmann-La Roche Ltd., Basel, Switzerland); Beckman enzymatic Cr reagent/Beckman AU5800 biochemical analyzer (Beckman Coulter, Inc., Brea, CA); Siemens enzymatic Cr reagent/Siemens RxL Max biochemical analyzer (Siemens, Bad Neustadt an der Saale, Germany); VITROS Chemistry Products CREA Slides (enzymatic)/VITROS 250 biochemical analyzer (Ortho Clinical Diagnostics, Johnson & Johnson, Rochester, NY); Maker enzymatic Cr reagent (Sichuan Maker Biotechnology Co., Ltd., Chengdu, China)/Beckman AU5800 biochemical analyzer; Merit Choice enzymatic Cr reagent (Merit Choice Bioengineering Co., Ltd., Beijing, China)/Siemens RxLMax biochemical analyzer; Leadman enzymatic Cr reagent (Leadman Biochemistry Co., Ltd., Beijing, China)/Hitachi 7180 biochemical analyzer (Hitachi, Tokyo, Japan); and Biosino enzymatic Cr reagent (Biosino Bio-Technology and Science Incorporation, Beijing, China)/Hitachi 7180 biochemical analyzer. The Beckman alkaline picrate Cr reagent was also evaluated on the Beckman AU5800 analyzer for comparison. All 8 SOE assays correlated well with the Beckman alkaline picrate method (Supplemental Table 1). A separate marker for renal function, cystatin C (CysC), was assayed by particle-enhanced turbidimetric assay (PETIA; Beijing Strong Biotechnologies, Inc., Beijing, China). The calcium dobesilate standard (≥98%) used in the in vitro experiments was purchased from Sigma Chemicals (St. Louis, MO) and the calcium dobesilate capsules taken by the volunteers and patients in in vivo experiments were manufactured by Xi’an Lijun Pharmaceutical Co., Ltd. (Xi’an, China).

### Subjects

Eighteen healthy participants (11 males and 7 females, 24–56 years of age) were recruited for in vivo interference experiments. They were divided into group I (5 males, 5 females) and group II (6 males, 2 females), and were advised to avoid the use of any other drugs and extreme exercise, to work regularly, and eat moderately throughout the experiment. Forty hospitalized patients being treated with calcium dobesilate (26 males and 24 females, 35–80 years of age) were recruited to study the clinical significance of calcium dobesilate interference. We did not perform any special interventions for the hospitalized patients. The study protocol was approved by the ethics committee of Peking Union Medical College Hospital (PUMCH). Written informed consent was given by all healthy participants and patients.

### In Vitro Interference Experiments

Calcium dobesilate interference was evaluated using a panel of 3 base serum pools prepared from nonicteric and nonhemolyzed samples obtained from patients receiving physical examinations or hospitalized at PUMCH in September 2014 who were free from calcium dobesilate. The Cr concentrations were 70, 133, and 442 μmol/L, determined according to the Clinical and Laboratory Standards Institute (CLSI) EP7-A2 guidelines.^15^ A calcium dobesilate standard was added to the base serum pools to prepare a dose-response series using the sequential mixing method as described in the CLSI EP7-A2 guidelines.^15^ The final calcium dobesilate concentrations in each of the 3 series were 0, 2, 4, 8, 16, 32, and 64 μg/mL. Cr levels were measured using the 8 SOE assays, as well as the Beckman alkaline picrate method. Specimens were analyzed in triplicate within 1 analytical run to obtain an average value. An internal quality control was applied during the experiments to ensure test quality.

Percent bias with respect to the different concentrations of calcium dobesilate was calculated relative to that of the drug-free specimen. Based on the biological variations and recommendations from clinical experts, a deviation for the Cr concentration of ±4% was considered clinically acceptable.^16^

### In Vivo Interference Experiments

Baseline serum samples from healthy participants in group I and II were collected prior to drug administration. Calcium dobesilate (500 mg) was given orally 3 times daily for 3 days to achieve a steady state according to pharmacokinetic information.^17^ Fasting blood samples were collected at trough drug levels on the morning of the 4th day (0 hours), followed by another 500 mg dose.

Blood samples of healthy participants in group I were collected at 1, 2, 3, 4, and 6 hours after drug administration. The Cr concentration of each sample was measured triplicately using the Roche SOE assay. CysC and calcium dobesilate concentrations were measured for each sample, and calcium dobesilate concentration was measured by a high performance liquid chromatography method at the Laboratory of Proteomics, Institute of Biophysics, Chinese Academy of Science. Ratios of Cr and CysC were calculated relative to the levels in the control serum for each participant. Alternatively, blood samples were collected at 2 hours after drug administration in participants of group II. The Cr concentration of each sample was then measured using 8 different SOE assays, as well as by the alkaline picrate assay.

### Interference in Patients

Forty serum or plasma samples remaining after clinical routine testing were collected from patients being treated with calcium dobesilate. For this, Cr concentrations were measured triplicately using SOE assays from Roche, Beckman, Maker, and Merit Choice and Beckman alkaline picrate assay within 1 analytical run, to obtain an average value. Calcium dobesilate and CysC concentrations were measured in each sample. Ten cases with Cr concentrations ranging from 42 to 326 μmol/L were used to compare values obtained by SOE assay and LC-IDMS/MS. LC-IDMS/MS was performed at the Beijing Hospital National Center for Clinical Laboratories, Ministry of Health (Beijing, China). Percent bias was calculated for the values derived from each SOE assay against those obtained by LC-IDMS/MS.

### Data Analysis

MedCalc Statistical Software (version 13.3.3, Broekstraat, Mariakerke, Belgium) and origin graphics software (version 7.5, Origin Lab Corp., Northampton) were used for graphics. Statistical analyses were performed using SPSS19.0 (SPSS Inc., Chicago, IL). Continuous data are presented as means and standard deviations or medians (range, interquartile range [IQR], 25%–75%] as appropriate. Paired *t* tests were used for all comparisons, with *P* < 0.05 defined as statistically significant.

## RESULTS

### Exogenous Calcium Dobesilate Interferes With Creatinine Quantification by Sarcosine Oxidase Assay

**FIGURE 1:**
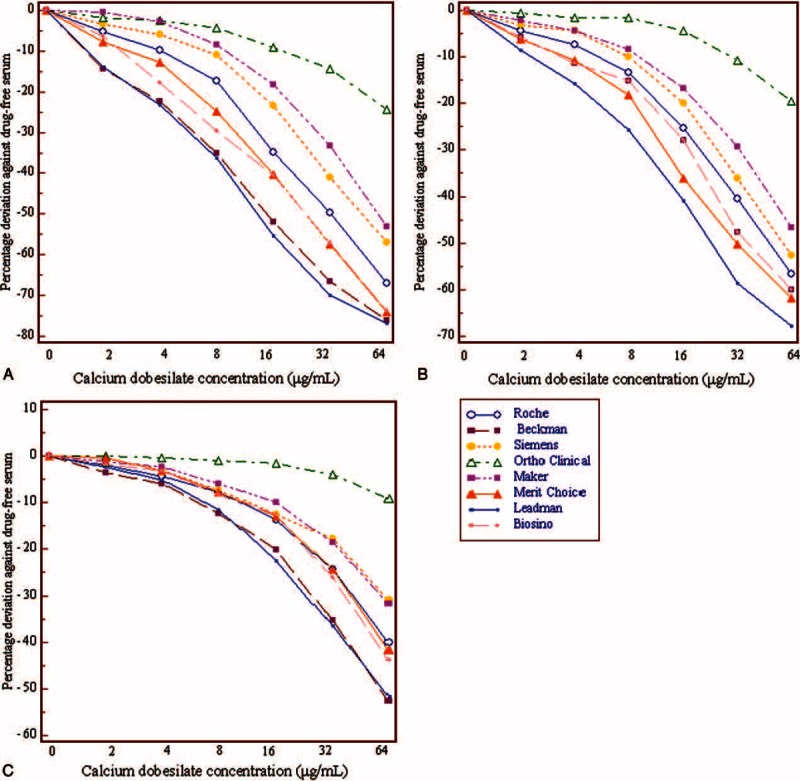
Effects of the exogenous addition of calcium dobesilate on creatinine (Cr) quantification in 8 sarcosine oxidase-based assay systems: Roche, Beckman, Siemens, Ortho Clinical, Maker, Merit Choice, Leadman, and Biosino. Panels A, B, and C show the data for basal Cr concentrations of 70, 133, and 442 μmol/L, respectively.

**FIGURE 2:**
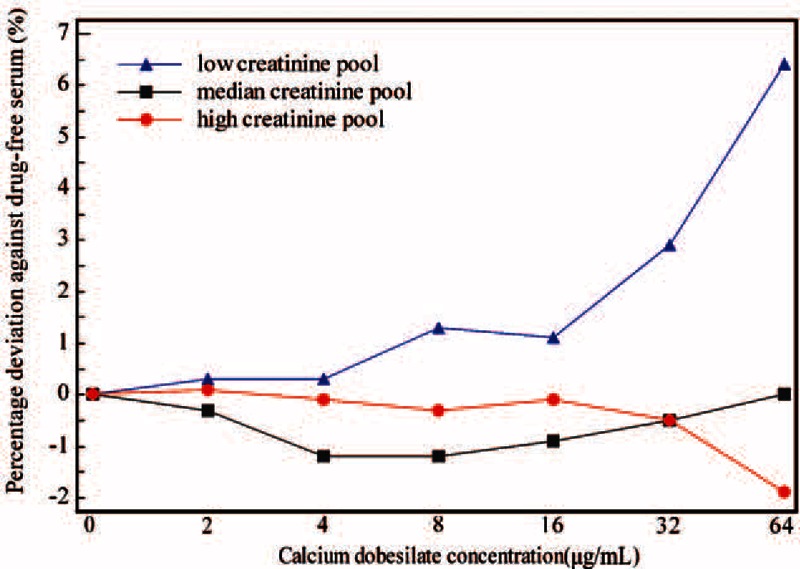
Effects of the exogenous addition of calcium dobesilate on Cr quantification in the Beckman alkaline picrate assay. The low, median, and high Cr pool shows data for basal Cr concentrations of 70, 133, and 442 μmol/L, respectively.

The exogenous addition of calcium dobesilate produced a negative dose-dependent effect on the Cr concentration in all 8 SOE assays examined (Figure 1). The discrepancies between the measured values from serum supplemented with 2 μg/mL calcium dobesilate and drug-free controls exceeded the clinically acceptable margin of error (−4%) at low and medium Cr levels Cr in the Roche, Beckman, Merit Choice, Leadman, and Biosino SOE assays. In the presence of 8 μg/mL calcium dobesilate, bias from drug-free serum ranged from −4.4% to −36.3% for the low-Cr level of interference samples in the 8 assays investigated (Figure 1A); for medium and high-Cr level of interference samples, all bias of 8 SOE assays except Ortho Clinical were exceeded the clinically acceptable margin of error (−4%) (Figure 1B and C). In addition, the assays showed clinically significant differences in Cr concentration with the same calcium dobesilate concentration in the interference samples. As presented in Figure 1, the Ortho Clinical SOE assay showed the smallest negative interference, whereas the Leadman enzymatic assay exhibited the largest negative interference. The degree of interference also correlated with the basal Cr concentration. As a control, we also assessed the effect of calcium dobesilate on Cr analysis by the alkaline picrate method and found no notable interference with serum concentrations less than 32 μg/mL (Figure 2); however, a slight positive interference (6.4%) was observed at low Cr levels once the calcium dobesilate serum concentration reached 64 μg/mL.

### Oral Administration of Calcium Dobesilate Artificially Suppresses Creatinine Levels in Sarcosine Oxidase-based Assay

**FIGURE 3:**
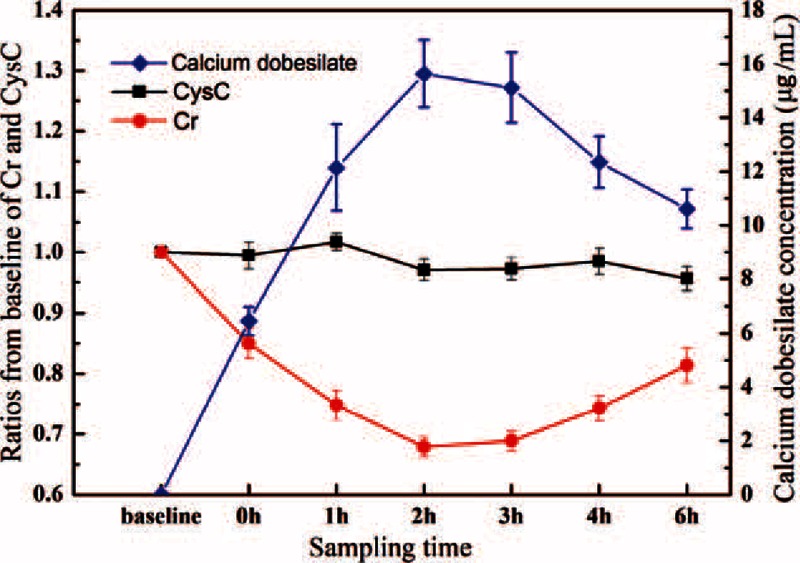
Changes in creatinine (Cr), cystatin C (CysC), and calcium dobesilate concentrations in healthy participants of group I before and after calcium dobesilate administration at various time points. Cr (μmol/L), CysC (mg/L), and calcium dobesilate (μg/mL) concentrations were measured by Roche sarcosine oxidase-based assay, particle-enhanced turbidimetric assay or HPLC, respectively. All data are shown as the mean ± SE. HPLC = high performance liquid chromatography.

Figure 3 illustrates the changes in the serum Cr, CysC, and calcium dobesilate values observed in healthy participants of group I before and after calcium dobesilate administration. After 3 days of administration, average trough calcium dobesilate concentrations (0 hours) were 6.64 (range, 2.66–8.33; IQR, 5.64–7.86) μg/mL, resulting in a decrease in Cr values of −4.6% to −17.0% relative to baseline control samples. After administration of an additional 500 mg of calcium dobesilate, peak calcium dobesilate concentrations in serum were 15.00 (range, 12.83–23.15; IQR, 13.63–19.67) μg/mL at 2 or 3 hours after administration. We also found that Cr values decreased with increasing drug concentration, with the lowest Cr levels obtained at 2 or 3 hours after drug administration (−28.3% to −40.9% relative to baseline), which increased thereafter. Moreover, no significant variation was observed in the level of CysC for participants throughout the experiment.

### Effects of Calcium Dobesilate are Assay-dependent

**FIGURE 4:**
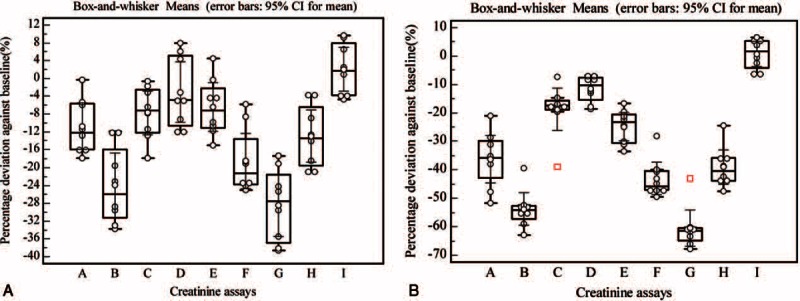
Box-and-whisker plot showing the percent deviation plots for creatinine concentrations at 0 and 2 hours after drug administration from healthy participants of group II against baseline. Creatinine concentrations were measured using 8 different sarcosine oxidase-based assays and 1 alkaline picrate assay. (A) Roche, (B) Beckman, (C) Siemens, (D) Ortho Clinical, (E) Maker, (F) Merit Choice, (G) Leadman, (H) Biosino, and (I) Beckman alkaline picrate assay. Panels A and B show data for at 0 and 2 hours after drug administration, respectively. The central boxes represent the interquartile range (IQR). The lines inside the boxes show the median and 95% confidence interval for each method. Whiskers extend from the minimum to the maximum value, excluding outliers (defined as values that exceed the upper or lower quartile ±1.5 × IQR).

The observable effects of calcium dobesilate administration exhibited interassay variation (Figure 4). Calcium dobesilate concentrations observed in healthy participants of group II were 4.79 (range, 2.72–9.55; IQR, 4.44–7.11) μg/mL and 18.53 (range, 7.03–20.21; IQR, 15.29–19.71) μg/mL at 0 and 2 hours, respectively. In contrast, no significant deviations were observed in serum Cr levels at these time points when measured by the Beckman alkaline picrate method (paired *t* test, *P* = 0.38 and 0.71 for 0 and 2 hours, respectively), with the average deviation of 2.1% and 0.7%, respectively (Figure 4). However, the Cr concentrations measured by 8 SOE assays at 0 and 2 hours all showed a significant difference with the baseline values (paired *t* test, *P* < 0.05). All Cr concentrations measured at 0 hours were artificially suppressed by calcium dobesilate with the exception of Ortho Clinical, with average deviations ranging from −28.5 % to −6.4% (Figure 4A). These effects were amplified at 2 hours, with average deviations ranging from −60.5 % to −11.6% (Figure 4B).

### Clinical Significance of Calcium Dobesilate Interference in Creatinine Analysis

The calcium dobesilate and CysC concentrations in the 40 specimens from patients taking calcium dobesilate were 11.92 (range, 1.27–63.35; IQR, 5.45–19.22) μg/mL and 1.74 (range, 0.70–5.58; IQR, 1.10–3.17) mg/L, respectively. Their Cr levels measured by Roche, Beckman, Maker, and Merit Choice enzymatic assays were 120.12 ± 70.85, 97.55 ± 62.74, 127.85 ± 77.90, 106.76 ± 66.58 μmol/L, respectively, which were far below that determined by Beckman alkaline picrate method (159.07 ± 93.93 μmol/L). Significant differences were observed between the Cr values obtained using the 4 SOE assays when compared to those from LC-IDMS/MS (Table 1). Specifically, the values measured by the enzymatic assays from Roche, Beckman, Maker, and Merit Choice showed average deviations of −20.0%, −22.4%, −14.2%, and −29.6%, respectively.

**TABLE 1:**
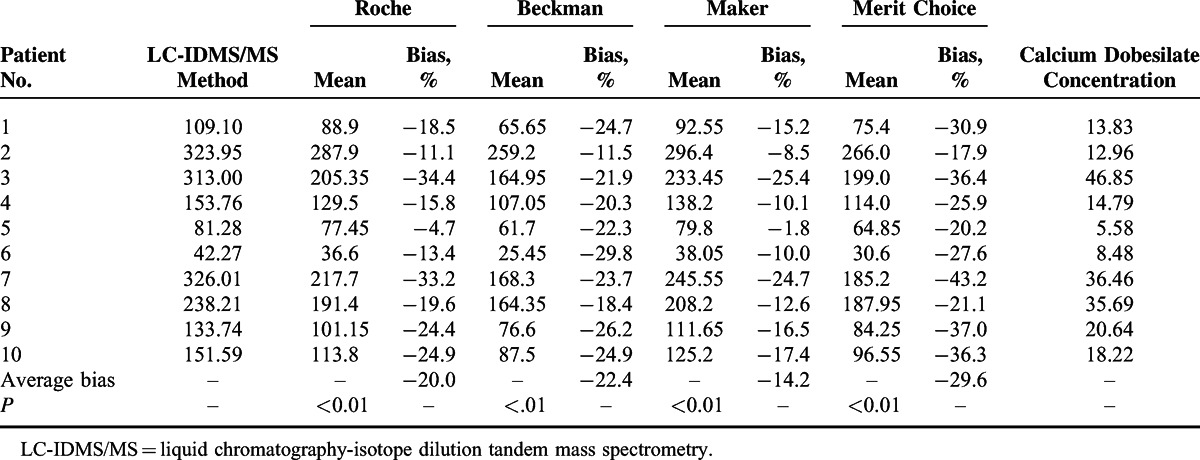
Relative Bias of Creatinine Values Measured by the Sarcosine Oxidase-Based Creatinine Assays of Roche, Beckman, Maker, and Merit Choice Against the LC-IDMS/MS Method. Creatinine is Shown as μmol/L; Calcium Dobesilate Concentration is Shown as μg/mL; Paired *t* Tests Were Used for the Comparisons Between Different Sarcosine Oxidase Assays and Reference Method

## DISCUSSION

In our study, calcium dobesilate was confirmed to negatively interfere with 8 sarcosine oxidase-based methods as determined by both in the in vitro and in vivo experiments, albeit to different degrees. Notably, the in vitro analyses revealed that calcium dobesilate interfered with all 8 sarcosine oxidase-based Cr assays in a dose-dependent manner at serum concentrations greater than 8 μg/mL. Calcium dobesilate is believed to remain mainly in its original form after excretion through the kidney and intestinal tract.^18^ Based on pharmacokinetic studies, the peak blood concentration of calcium dobesilate (8 μg/mL) is achieved at 6 hours after a single oral administration of 500 mg.^17,18^ Since the current recommended dosage of calcium dobesilate is 500 mg 3 times per day, its blood serum concentration is estimated to be 15 μg/mL at steady state.^17^ In our in vitro experiments, the blood serum concentrations of calcium dobesilate in 18 participants ranged from 2.66 to 9.55 μg/mL and 7.04 to 23.15 μg/mL at trough and peak concentrations, respectively. Nonetheless, concentrations present in the serum samples collected at the drug's projected trough were still high enough to cause an artificially lower Cr value as measured by SOE assay (Figures 3 and 4). However, participants showed the highest drug concentration at 2 or 3 hours after drug administration, which is inconsistent with previous reports showing that calcium dobesilate achieves a peak blood concentration 6 hours after administration.^17^ The discrepancy may have arisen because of differences in dosage forms or variation among individuals. In addition, calcium dobesilate is primarily excreted through the kidneys. Patients with renal inadequacy have exceedingly lower glomerular filtration rates and are thus prone to drug accumulation, which may result in a more severe interference. In our study, the highest calcium dobesilate concentration observed in patients was 63.35 μg/mL. In clinical practice, differences in the blood collection time and the analytical system used for Cr quantification may result in larger variations that could considerably confound clinical diagnoses.

Drug interference in biochemical analyses is a common, but often neglected, problem in routine clinical laboratory screenings. We have recognized the existence of this interference several times because of the different methods and systems used for Cr detection in our laboratory; however, potential sources of interference are nearly impossible to detect when only 1 method is used for analyses. The negative interference of calcium dobesilate with Cr measurement obtained by SOE assays would likely result in false kidney function evaluations, and thus conceal the extent of diabetic nephropathy in patients. For this reason, extra precautions should be taken to avoid false-negative results in Cr quantification using SOE assays in clinical practice.

As revealed by our statistics, 13,599 patients underwent calcium dobesilate therapy at Beijing Union Medical College Hospital in 2014, emphasizing the critical nature of this issue. CysC is a common, potentially more sensitive parameter for renal function assessment,^19^ and levels are unaffected by calcium dobesilate as it is tested by PETIA. Therefore, we recommend that CysC be used to assess renal function in patients treated with calcium dobesilate.

Although the negative interference of calcium dobesilate in SOE Cr methods has been observed for nearly 30 years, the issue remains unresolved. Is the cost necessary to eliminate interference too high, or is it still technically difficult to solve the problem? While the mechanism of interference has not been entirely explored yet, we have generated an initial, chemically-based hypothesis. First, the assay is based on the enzymatic degradation of Cr and its reaction products by creatininase, creatinase, and sarcosine oxidase. The hydrogen peroxide produced by the oxidation of sarcosine is determined using a modified Trinder reaction. The fundamental structure of calcium dobesilate is a hydroquinone ring, which may consume the hydrogen peroxide produced by the peroxidase-indicating system during the reaction to subsequently give rise to negative interference. Alternatively, we hypothesize that calcium dobesilate may react with other constituents of the Trinder reaction, therefore interfering with chromophore formation. Furthermore, it has been speculated that calcium dobesilate might affect the stability of the chromophore produced (ie, reduce the quinoneimine dye). Interestingly, significant differences in the degree of interference were observed among the 8 SOE assays investigated in our study. We speculate this may be due to the distinct sample or reagent volumes, or variations in the final calcium dobesilate concentrations, hydrogen peroxide production, or the structural characteristics of chromogen used in each assay system. An understanding of these exact mechanisms may facilitate the introduction of suitable approaches for eliminating or minimizing calcium dobesilate interference.

The strengths of the present study are as follows. First, the LC-IDMS/MS method was adopted to measure Cr as the reference method to evaluate interference using patient specimens. This approach more objectively reflects the presence of interference than those using the alkaline picrate method, as previously compared in the other study.^5^ Second, blood concentrations of calcium dobesilate in healthy participants and patients were measured, which would have consolidated our results of dose-dependent deviations in Cr levels. However, certain limitations were still present in our analyses and include a relatively small sample size and a significant age difference between healthy participants and hospitalized patients, which may influence the results. Third, we did not perform any special interventions for the hospitalized patients, and failed to record their administration time. In addition, some sources of potential bias or imprecision should be considered, such as random errors in detection, and additional rounds of freezing and thawing for samples examined by LC-IDMS/MS and high performance liquid chromatography.

In conclusion, both in vitro and in vivo analyses revealed that calcium dobesilate produced a significant negative interference on Cr concentration in sarcosine oxidase-based assays, which may result in false kidney function evaluations. Clinically significant differences in the degree of interference were observed among the assays. To limit these issues, manufacturers should strive to eliminate this interference, medical technologists should strengthen communication with physicians, and extra care should be taken in evaluating the Cr levels in patients treated with calcium dobesilate.
